# Anderson-Fabry disease in heart failure

**DOI:** 10.1007/s12551-018-0432-5

**Published:** 2018-06-16

**Authors:** M. M. Akhtar, P. M. Elliott

**Affiliations:** Institute of Cardiovascular Science, University College London, London, UK

**Keywords:** Anderson-Fabry disease, Globotriaosylceramide, *GLA* gene, Heart failure

## Abstract

Anderson-Fabry disease is an X-linked lysosomal storage disorder caused by mutations in the GLA gene that result in deficiency of the enzyme alpha-galactosidase A. The worldwide incidence of Fabry’s disease is reported to be in the range of 1 in 40,000–117,000, although this value may be a significant underestimate given under recognition of symptoms and delayed or missed diagnosis. Deficiency in alpha-galactosidase A causes an accumulation of neutral glycosphingolipids such as globotriaosylceramide (Gb3) in lysosomes within various tissues including the vascular endothelium, kidneys, heart, eyes, skin and nervous system. Gb3 accumulation induces pathology via the release of pro-inflammatory cytokines, growth-promoting factors and by oxidative stress, resulting in myocardial extracellular matrix remodelling, left ventricular hypertrophy (LVH), vascular dysfunction and interstitial fibrosis. Cardiac involvement manifesting as ventricular hypertrophy, systolic and diastolic dysfunction, valvular abnormalities and conduction tissue disease is common in AFD and is associated with considerable cardiovascular morbidity and mortality from heart failure, sudden cardiac death and stroke-related death.

## Introduction

Anderson-Fabry disease (AFD) is an X-linked lysosomal storage disorder caused by mutations in the *GLA* gene that result in deficiency of the enzyme α-galactosidase A. The worldwide incidence of Fabry’s disease is reported to be in the range of 1 in 40,000–117,000, although this value may be a significant underestimate given under recognition of symptoms and delayed or missed diagnosis (Zarate and Hopkin 2008; Mehta et al. 2004). The prevalence in selected patient cohorts is even higher and reported to be between 0.25–3.5% in male haemodialysis patients, 0.9–3.9% in male patients with hypertrophic cardiomyopathy (HCM) and 3–5% in patients with cryptogenic stroke (Linhart and Elliott 2007; Sachdev et al. 2002; Nakao et al. 2003; Kotanko et al. 2004; Kubo et al. 2017; Shi et al. 2014; Doheny et al. 2018; Elliott et al. 2011; Monserrat et al. 2007; Hagege et al. 2011).

Deficiency in α-galactosidase A causes an accumulation of neutral glycosphingolipids such as globotriaosylceramide (Gb3) in lysosomes within various tissues including the vascular endothelium, kidneys, heart, eyes, skin and nervous system (Clarke 2007). Gb3 accumulation induces pathology via the release of pro-inflammatory cytokines, growth-promoting factors and by oxidative stress, resulting in myocardial extracellular matrix remodelling, left ventricular hypertrophy (LVH), vascular dysfunction and interstitial fibrosis (Putko et al. 2015; Linhart 2006; Moon et al. 2003). Lysosomal storage deposits also reduce the activity of respiratory chain enzymes I, IV and V and reduce cellular levels of energy-rich phosphates (Lucke et al. 2004).

Cardiac involvement manifesting as ventricular hypertrophy, systolic and diastolic dysfunction, valvular abnormalities and conduction tissue disease is common in AFD and is associated with considerable cardiovascular morbidity and mortality from heart failure, sudden cardiac death and stroke-related death (O'Mahony and Elliott 2010; Patel et al. 2015).

## Genetics

The *GLA* gene, located at Xq22.1, comprises seven exons over 12 Kb and encodes a 101 kD homodimeric glycoprotein (Linhart and Elliott 2007; Kornreich et al. 1989). In excess of 700 predominantly missense (60%), mutations in *GLA* have been identified (Schaefer et al. 2005; Shabbeer et al. 2006). Deletion of several exons or even the entire gene is uncommon and may result in a negative genetic test particularly in heterozygote females unless multiplex ligation-dependent probe amplification or copy number variation is analysed (Schirinzi et al. 2008). Most mutations in *GLA* affect protein-folding by disrupting the hydrophobic core of the *GLA* protein or by altering active binding sites and also affect enzyme localisation to the lysosome and result in reduced enzyme activity (Linhart and Elliott 2007; Garman 2007).

Many of the pathogenic mutations in *GLA* are ‘private’, and this limits the ability to undertake detailed genotype-phenotype analysis. In women, random X-chromosome inactivation (lyonization) means that α-galactosidase levels in plasma and white cells can fall within the normal range but disease expression still occurs, albeit later and generally less severe than in male hemizygotes (Dobrovolny et al. 2005). Severe disease in women may occur when there is skewed inactivation of X chromosomes in favour of the mutant allele (O'Mahony and Elliott 2010; The Human Gene Mutation Database 2018; Ries and Gal 2006; Echevarria et al. 2016). Inter- and intra-familial variation in phenotype may be modulated by other genetic modifiers, environmental factors and epigenetics although more research is being undertaken in this field (Ries and Gal 2006; Altarescu et al. 2005; Hassan et al. 2017; Cammarata et al. 2015; Rigoldi et al. 2014; Teitcher et al. 2008).

Some mutations in the *GLA* gene are associated with later-onset or ‘non-classical’ disease with a predominant or exclusive cardiac phenotype. These include variants such as N215S or R112H and are often referred to as ‘cardiac variants’ and the later-onset phenotype may be due to residual α-galactosidase activity (von Scheidt et al. 1991; Eng et al. 1993; Spada et al. 2006; Frustaci et al. 2001). Affected patients with ‘classical’ disease have a significantly higher rate of adverse events compared to those with ‘non-classical’ disease, and males with ‘classical’ disease tend to have a higher left ventricular mass and lower renal function than those with ‘non-classical’ forms of disease (Arends et al. 2017a). The non-classical N215S mutation is one of the most prevalent AFD mutations in Europe (Muntze et al. 2018). The later symptom onset, delayed development of LVH and the relative paucity of extra-cardiac manifestations means the cardiac phenotype often mimics non-obstructive HCM, which results in a significant delay in diagnosis in probands and delay in commencement of Fabry-specific therapy (Lavalle et al. 2018; Germain et al. 2018).

## Cardiac disease in AFD

### Histopathology

**Fig. 1:**
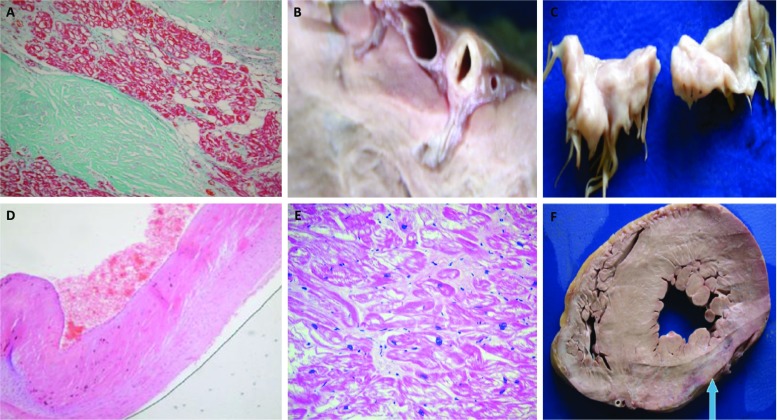
(Histological features of Anderson-Fabry disease). **a** Masson trichrome stain demonstrating vacuolization within myocytes with extension into the right bundle branch of the conduction pathway. **b** Section through the left circumflex coronary artery showing diffuse wall thickening without significant luminal occlusion. **c** Section through the mitral valve apparatus demonstrating mild mitral valve leaflet thickening and ballooning of the anterior and posterior valve leaflets. **d** H&E staining demonstrating hyaline pink material within the spongiosa and fibrosa. **e** H&E staining of the ventricular myocardium with evidence of myocyte hypertrophy and marked vacuolization of the cytoplasm of myocytes. **f** Transverse section of the heart. There is concentric left ventricular hypertrophy associated with thinning of the posterolateral wall (blue arrow). Mild right ventricular hypertrophy is also present and prominent in the posterobasal area of the RV. Fig. 1a–f: Reprinted from Cardiovasc Pathol.; 19(5), Sheppard MN, Cane P, Florio R, Kavantzas N, Close L, Shah J, Lee P, Elliott P; “A detailed pathologic examination of heart tissue from three older patients with Anderson-Fabry disease on enzyme replacement therapy.”; pages 293–301; 2010, reproduced with permission from Elsevier (Sheppard et al. 2010)

Histological analysis of hearts affected by AFD reveals accumulation of glycosphingolipids within cardiomyocytes, the cardiac conduction system (Fig. 1a) and the coronary vasculature (Fig. 1b). Glycosphingolipid accumulation also contributes to valve leaflet thickening (Fig. 1c, d) (Wu et al. 2010). There is marked vacuolization within the cytoplasm of cardiomyocytes (Fig. 1e), and electron microscopy demonstrates dense lamellar deposits of Gb3 within lysosomes. Myocardial disarray can be observed but is much less prominent than in patients with familial HCM (Putko et al. 2015; Sheppard 2011). Interstitial fibrosis may be evident with a subepicardial or mid-wall pattern of fibrosis that becomes more extensive in the posterolateral basal portion of the left ventricle (Fig. 1f) (Moon et al. 2003). Uncommonly, myocardial scarring is associated with aneurysm formation (Moon et al. 2003; Poulin et al. 2015).

### Clinical presentation

The classical variant of AFD usually affects men with very low or absent levels of serum α-galactosidase A. Patients develop extra-cardiac signs and symptoms such as angiokeratoma, gastrointestinal disturbance, hypohidrosis and peripheral neuropathy in the first decade of life and renal involvement with proteinuria and progression to end-stage renal failure are often established by the fourth decade of life (Mehta et al. 2004; Linhart and Elliott 2007; O'Mahony and Elliott 2010; Kubo 2017). The cardiac phenotype typically develops between the 3rd and 5th decade of life (Putko et al. 2015; Havndrup et al. 2010).

In heterozygous females, cardiac and extra-cardiac manifestations are generally milder and develop later in life than in hemizygous males (Kubo 2017). Women tend to have slower disease progression and a longer median cumulative survival than affected males (Mehta et al. 2004; Linhart and Elliott 2007).

As many of 10% of patients have a cardiac event as their index presenting symptom, and more than 60% of patients experience cardiovascular symptoms such as dyspnoea, angina, palpitations, syncope and peripheral oedema (Mehta et al. 2004; Clarke 2007; Wu et al. 2010; Eng et al. 2007). Dyspnoea is associated with left ventricular hypertrophy and is caused by left ventricular outflow tract obstruction, diastolic dysfunction and systolic dysfunction. Less commonly, patients with advanced disease develop symptomatic aortic or mitral valve disease. In the Fabry outcome survey, exertional dyspnoea occurred in 23% of affected males and females and as many as 10% developed advanced heart failure symptoms (Putko et al. 2015) (Patel et al. 2015; Linhart et al. 2007).

Cardiac disease is the major cause of death in men and women with AFD, accounting for 38% of all-cause mortality (Mehta et al. 2009a). The prevalence of cardiovascular death is reported to be 3% (annual incidence of 0.52 per 100 person-years) in a large observational study of patients over a mean follow-up period of 7.1 years in which 2.4% of the overall cohort had a sudden cardiac death and 0.97% suffered a heart failure-related mortality (Patel et al. 2015).

### Left ventricular hypertrophy and LV outflow tract obstruction

Left ventricular hypertrophy (LVH) is the commonest structural cardiac abnormality observed (Linhart et al. 2007). Forty percent of AFD patients have LVH at diagnosis but this proportion rises to 77% in those over the age of 75 (Linhart et al. 2000; Lidove et al. 2016). In heterozygous females, plasma α-galactosidase levels do not correlate with the severity of LVH. LVH is caused by intra-cellular accumulation of Gb3 as well as by the release of hypertrophy-inducing growth factors and extracellular matrix remodelling. This is supported by experimental studies demonstrating that plasma from affected patients induces rat vascular smooth muscle cell and neonatal mouse cardiomyocyte proliferation in culture when compared to cell culture using plasma from normal controls or from hypertensive-control populations (Barbey et al. 2006a). Another study identified the presence of a plasma proliferative factor, sphingosine-1 phosphate (S1P), and demonstrated that plasma levels of S1P correlated with left ventricular mass index and common carotid artery intima-media thickness in patients with AFD (Brakch et al. 2010). S1P-treated mice also developed cardiovascular remodelling with cardiac hypertrophy similar to that seen in affected AFD patients (Brakch et al. 2010).

ECG abnormalities indicative of LVH include large voltage QRS complexes, and repolarisation abnormalities which are present in 61% of men and 18% of women over the age of 30 (Linhart et al. 2000). ECG changes may predate abnormal LV morphology on cardiac imaging (Linhart et al. 2000).

**Fig. 2:**
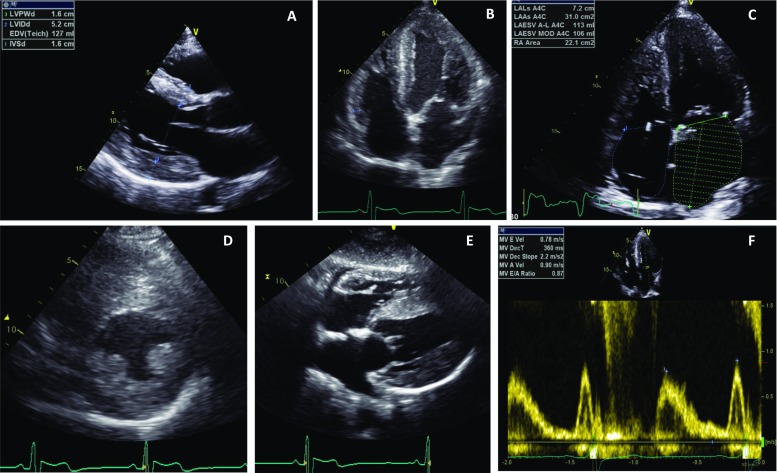
(Echocardiogram features of Anderson-Fabry disease). **a** Transthoracic echocardiogram (TTE) parasternal long-axis view (PLAX) demonstrating concentric LVH with an interventricular septum (IVS) and left ventricular posterior wall (LVPW) diameter of 16 mm. **b** Apical 4 chamber view on TTE demonstrating the presence of concentric LVH. **c** Moderately dilated left atrium (31 cm^2^) on apical 4-chamber TTE view. **d** TTE parasternal short-axis view (PSAX) at mid-ventricular level demonstrating concentric LVH and hypertrophied papillary muscles. **e** TTE subcostal view demonstrating concentric LVH. **f** Pulsed wave Doppler across mitral valve inflow demonstrating impaired relaxation (grade 1 diastolic dysfunction) with E:A ratio of 0.87

The prevalence of LVH increases with age in patients treated and untreated with enzyme replacement therapy (ERT). Concentric LV remodelling precedes overt left ventricular hypertrophy, but when hypertrophy is established, it can be clinically indistinguishable from other causes of left ventricular hypertrophy including hypertrophic cardiomyopathy (HCM) (Putko et al. 2015; Linhart et al. 2000). Other causes for concentric LV remodelling and hypertrophy include hypertension, aortic valve disease or cardiac amyloidosis. The LVH pattern in AFD can be asymmetric, eccentric, distal or concentric although a concentric pattern of hypertrophy is the most prevalent (Fig. 2a–e) (Wu et al. 2010; Kampmann et al. 2008). 2.2% of patients develop asymmetric septal hypertrophy with a septal to posterior wall dimension ratio > = 1.5 (Wu et al. 2010). Right ventricular hypertrophy (RVH) also develops in as many as 25% of patients with a similar prevalence in men and women (Wu et al. 2010; Palecek et al. 2008).

There is an inverse relationship between left ventricular mass and estimated glomerular filtration rate (eGFR) and a positive correlation between LV mass and the common carotid intima-medial thickness (Linhart et al. 2007; Barbey et al. 2006a). LVH is associated with an increased frequency of dysrhythmia. The presence of increased cardiovascular mass at baseline evaluation is associated with an increased event rate over follow-up (Wu et al. 2010; Arends et al. 2017b). In a multivariate model of patients naïve to ERT, the presence of LVH significantly increased the odds of cardiovascular events by a factor of 4.8 in males (odds ratio 4.8; 95% CI 1.0–22.2; *p* = 0.046) and 8.2 (odds ratio 8.2; 95% CI 2.6–26.0; *p* < 0.001) in females (Patel et al. 2011).

Left ventricular volume, LV dimension, stroke volume and LV ejection fraction (LVEF) are within the normal range in most patients, but left ventricular and papillary muscle hypertrophy can cause a reduction in LV cavity size as well as contribute to the development of left ventricular outflow tract obstruction (LVOTO) or mid-cavity obstruction (MCO) which exacerbate symptoms of chest pain, dyspnoea and syncope (Sachdev et al. 2002; Linhart et al. 2000). Resting LVOTO is rare but may be provocable in approximately 50% of patients with LVH (Calcagnino et al. 2011).

### Exercise physiology

Exercise testing in affected patients demonstrates an increase in stroke volume on exercise, albeit less than predicted from normal population data (Lobo et al. 2008). In those with a more advanced cardiac phenotype (impaired diastolic function and higher LV mass), there is a reduction in end-diastolic volume and a lack of reduction in end-systolic volume on exercise resulting in evidence of abnormal stroke volume augmentation (Spinelli et al. 2008). There is also evidence that in a significant proportion of patients (46%), there is a diastolic blood pressure drop on exercise (Bierer et al. 2005). Use of ERT results in a mild improvement in anaerobic threshold value, but only over the first year of therapy (Lobo et al. 2008; Bierer et al. 2006).

### Myocardial tissue characterisation with magnetic resonance imaging

**Fig. 3:**
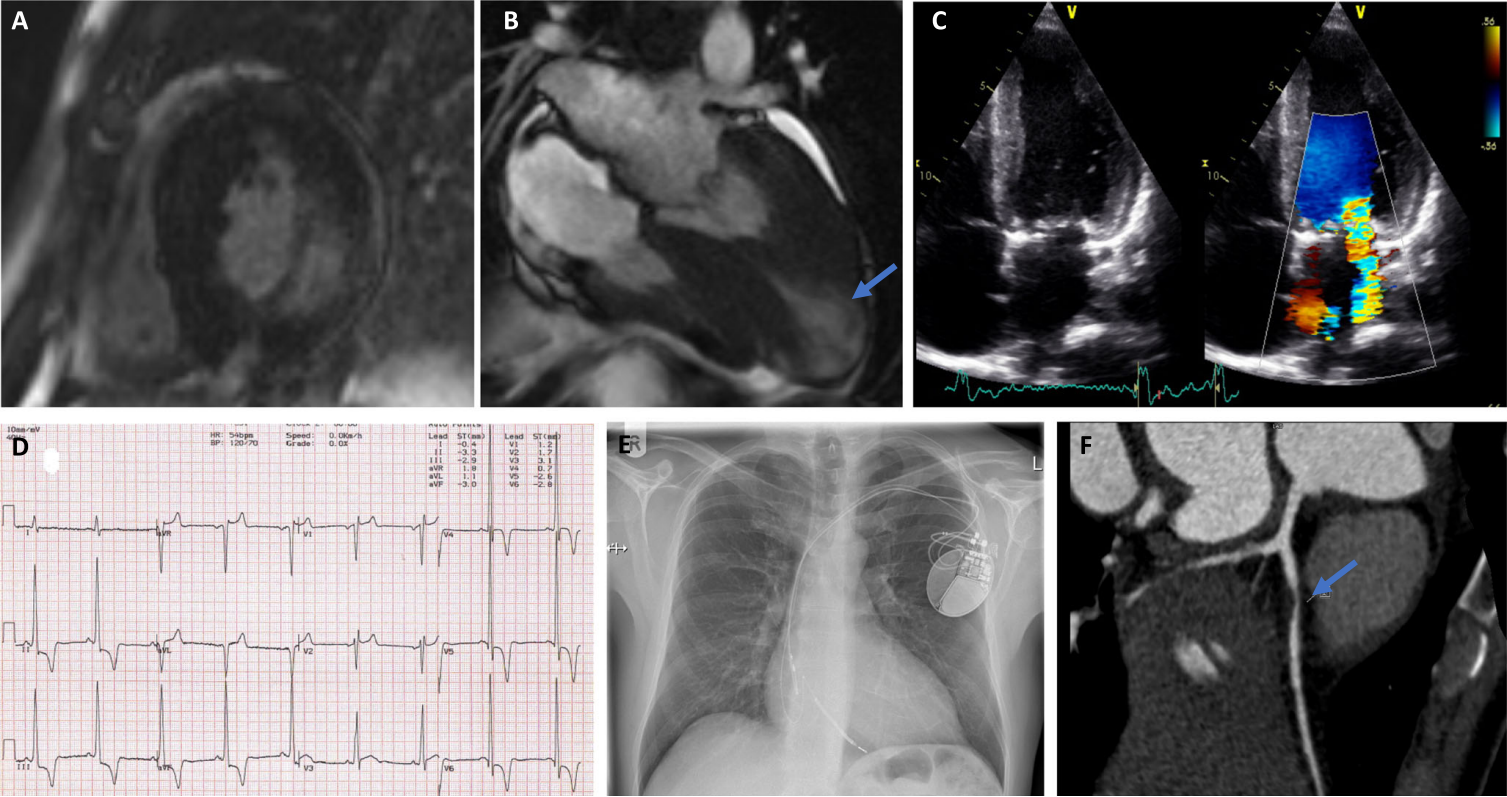
(Complications of Anderson-Fabry disease). **a** Mid-LV short-axis view on cardiac MRI (CMRI) showing concentric LVH with dense subepicardial and mid-wall LGE in the inferolateral wall. **b** Apical left ventricular aneurysm (blue arrow) demonstrated on a 4-chamber view on CMRI. **c** TTE apical 4-chamber view demonstrating severe eccentric (posteriorly) directed jet of MR due to mitral valve thickening and dysfunction. **d** 12-Lead electrocardiogram of a patient with conduction disease—borderline RBBB (QRS duration 120 ms) and right axis deviation. There is also presence of large voltage QRS complexes (prominent R waves V4-6) and repolarisation abnormalities with T wave inversion inferolaterally (leads II, III, aVF and V4-6) meeting ECG criteria for LVH. **e** Chest X-ray demonstrating a dual chamber secondary prevention implantable cardioverter-defibrillator (ICD) in an AFD patient presenting with an out-of-hospital cardiac arrest. **f** CT coronary angiogram of an AFD patient symptomatic with chest pain, demonstrating a non-calcified proximal LAD plaque lesion (blue arrow) associated with severe stenosis (70–99% diameter reduction)

Cardiac MRI with gadolinium contrast administration often identifies characteristic subepicardial basal to mid-inferolateral late gadolinium enhancement (Fig. 3a), which in the setting of advanced disease with a ‘burnt out’ phenotype of LV systolic dysfunction may be associated with hypokinesia or thinning of the basal posterior wall (Takenaka et al. 2008). Glycosphingolipid intra-myocardial accumulation can shorten the non-contrast T1 mapping parameter on cardiac magnetic resonance imaging. Septal T1 values are generally low in AFD and increased in other diseases causing LVH such as hypertension, HCM, aortic stenosis and AL cardiac amyloidosis except in regions of extensive myocardial replacement fibrosis, and consequently, it can be a useful imaging parameter in patients with unexplained LVH undergoing cardiac MRI (Sado et al. 2013; Linhart and Cecchi 2018). T1 values correlate inversely with maximal LV wall thickness in AFD (Sado et al. 2013).

Recently, high-sensitivity troponin (hs-TnT) has been shown to positively correlate with late gadolinium enhancement, and elevated hs-TnT levels are associated with a reduction in LVEF over follow-up suggesting that it may be a useful biomarker for progression in Fabry’s cardiomyopathy (Seydelmann et al. 2016).

### Diastolic dysfunction and restrictive physiology

Imaging modalities including pulsed wave Doppler and tissue Doppler imaging may be used in the echocardiographic assessment of diastolic function in AFD patients although more sensitive markers include global diastolic strain rate analysis. Diastolic dysfunction is common and results in impaired ventricular filling due to increased ventricular stiffness and impaired relaxation (Fig. 2f). Diastolic dysfunction is probably the commonest cause of exertional dyspnoea and reduced exercise tolerance in patients and may be present before the development of overt LVH (Pieroni et al. 2003). In general, diastolic dysfunction progresses in parallel with ventricular hypertrophy, but rarely, patients can develop severe diastolic dysfunction with a restrictive filling pattern or rising pulmonary pressures that is associated with a poor prognosis. NT-proBNP levels demonstrate a significant positive correlation with LV diastolic dysfunction and are a sensitive marker in detecting early cardiac changes (Torralba-Cabeza et al. 2011).

Diastolic dysfunction contributes to left atrial enlargement which in turn predisposes to atrial arrhythmias including atrial fibrillation (Fig. 2c) (Wu et al. 2010; Weidemann et al. 2003). Left atrial enlargement, increased left atrial stiffness index and reduced atrial compliance also occurs, even in the absence of LVH suggesting an impact on atrial myocardial function and properties early in the disease phase (Boyd et al. 2013). Left atrial volume is usually less dilated than in patients with HCM but speckle tracking demonstrates an atrial cardiomyopathy in Fabry affecting the three phasic functions of the left atrium (Saccheri et al. 2018). This impact on atrial size and function may be due to raised LV filling pressures passively transmitted to the left atrium or due to glycosphingolipid deposition in the left atrial myocardium associated with interstitial fibrosis (Chimenti et al. 2010). Over a third of patients on ERT have evidence of left atrial enlargement (Wu et al. 2010).

### Systolic dysfunction

Left ventricular systolic dysfunction is a less common complication of Fabry’s cardiomyopathy. The prevalence of LV systolic dysfunction amongst patients with AFD is 6–8% which is associated with an increased overall incidence of heart failure-related mortality (Wu et al. 2010; Rosmini et al. 2017). Left ventricular systolic dysfunction may develop as the disease progresses, in particular in ERT-untreated patients, and may occur in part due to progressive LV myocardial fibrosis (Shah et al. 2005a). In a study of patients on ERT, only a small proportion of patients, all males (6.5%), developed LV systolic dysfunction and a couple of individuals also developed LV dilatation, including one with a LV aneurysm (Fig. 3b) (Wu et al. 2010).

Tissue Doppler imaging (TDI) helps to identify early subclinical cardiac involvement before the onset of left ventricular hypertrophy or systolic and diastolic impairment (Pieroni et al. 2003; Pieroni et al. 2004). Strain and strain rate analysis are also useful in identifying patients with reduced myocardial function at an early phase (Shanks et al. 2013; Gruner et al. 2012).

LV basal posterior wall thinning with progressive fibrosis is significantly associated with progression to advanced heart failure and cardiac death (Kawano et al. 2007; Kampmann et al. 2002).

### Valvular heart disease

Gb3 accumulation occurs on both left- and right-sided valves although clinically relevant disease is mostly confined to the aortic and mitral valves possibly due to the greater haemodynamic stress (Putko et al. 2015; Linhart 2006). Individuals can develop valvular stenosis, regurgitation or prolapse (Desnick et al. 1976). Mild thickening of the left-sided valves is seen in as many as a quarter of patients (Sheppard 2011). Mitral valve dysfunction may be accentuated by papillary muscle hypertrophy and by systolic anterior motion of the mitral valve leaflet resulting in altered coaptation and an eccentric jet of mitral regurgitation (Fig. 3c). There is no association between the presence of valvular abnormalities and α-galactosidase levels. (Wu et al. 2010).

Although the presence of valvular disease is common, few patients develop valvular regurgitation or stenosis sufficient to warrant surgical intervention with valve repair or replacement (Wu et al. 2010; Choi et al. 2009; Fernandez et al. 2012). There are reports of successful valvular intervention including using newer transcatheter aortic valve replacements (TAVR) in patients with Fabry’s disease (Giustino et al. 2014).

### Conduction disease and arrhythmias

The ECG in AFD is often abnormal. Apart from QRS voltage changes, up to 40% of affected male patients have a short PR interval typically without the presence of a concomitant delta wave suggesting accelerated AV nodal connection (Pochis et al. 1994; Jastrzebski et al. 2006). With increasing age, patients may develop conduction disease with a progressively prolonged PR interval and a broadened QRS duration. A prolonged QRS duration (> 120 ms) as well as abnormal QRS axis is seen in 9% of patients (Fig. 3d) (O’Mahony et al. 2011).

AFD is associated with atrial and ventricular arrhythmias (Frustaci and Chimenti 2007). Resting bradycardia and an impaired heart rate response on exercise are very frequent (Lobo et al. 2008). Bradyarrhythmias resulting from sinus node disease and atrioventricular block may necessitate permanent pacemaker implantation, and the rates of anti-bradycardia pacing are more than 25 times greater than that in the general population (O’Mahony et al. 2011). PR interval duration and QRS duration are independent predictors of the need for pacemaker implantation (O’Mahony et al. 2011). In those with an intra-cardiac device in situ, there is a high rate of atrial or ventricular pacing (Acharya et al. 2012). The mechanism for premature conduction disease may be related to autonomic dysfunction as well as degeneration of the cardiac conduction system with glycosphingolipid accumulation, apoptosis and vacuolation (Frustaci and Chimenti 2007; Ikari et al. 1992; Mehta et al. 2010).

Affected individuals are prone to tachyarrhythmias including atrial fibrillation (AF) due to left atrial enlargement and diastolic dysfunction, non-sustained ventricular tachycardia (NSVT) or sustained malignant ventricular arrhythmia such as ventricular tachycardia (VT) or ventricular fibrillation (VF). The frequency of AF or NSVT in studies vary and reflect differences in cohort characteristics and the method of arrhythmia monitoring. In a study of 60 Fabry patients undergoing 24-h Holter analysis, 3.9% had persistent AF and 13.3% had paroxysmal AF. Age was an independent predictor for AF (odds ratio 1.2, 95% CI 1.1–1.3, *p* = 0.001) (Shah et al. 2005b). Glycosphingolipid deposition in the atrial myocardium, atrial dilatation due to diastolic dysfunction and elevated LV filling pressure increase the risk of AF (Linhart et al. 2007). AF may contribute to the increased risk of stroke in addition to AFD-related small vessel cerebrovascular disease.

NSVT occurs mostly in males with moderate to severe hypertrophy (Shah et al. 2005b). The clinical significance of NSVT is yet to be determined, but it is an established risk factor for sudden cardiac death (SCD) in other hypertrophic disorders. In one study, sudden cardiac death events only occurred in patients with prior documentation of non-sustained ventricular tachycardia and with late gadolinium enhancement on cardiac MRI (Weidemann et al. 2013). When implantable loop recorder (ILR) implants are used to assess for arrhythmias instead of conventional 24-h Holter, over 30% of patients have VT episodes (NSVT or sustained VT) and a similar percentage were identified to have short runs of paroxysmal AF lasting more than 3 min (Weidemann et al. 2016). Other series have identified ventricular arrhythmias in 14% of affected males and 20% in affected females (Pinderski and Strotmann 2006).

Cases of sudden cardiac death (SCD) are attributed to malignant bradyarrhythmias or tachyarrhythmia but are rarely recorded (Frustaci and Chimenti 2007; Eckart et al. 2000; Carter et al. 1995; Sivaloganathan 1992). The incidence of SCD is difficult to accurately assess due to small patient numbers and the rarity of the condition. Single-centre experience reports an annual incidence of SCD between 0.3–1.4% (Patel et al. 2015; Shah et al. 2005b; Baig et al. 2017). In patients in whom there is felt to be a sufficiently elevated risk of sudden cardiac death, an implantable cardioverter-defibrillator may be implanted to protect against malignant ventricular arrhythmias (Fig. 3e).

A recent systematic review identified that SCD is a major cause for cardiovascular mortality in AFD patients accounting for 62% of reported deaths. In this review, the average prevalence of ventricular tachycardia was 15.3% and risk factors associated with SCD were age > 40 years, male gender, left ventricular hypertrophy, NSVT and presence of late gadolinium enhancement on CMRI (Baig et al. 2017).

### Vascular disease

Glycosphingolipid deposits are found in in the endothelium and media of blood vessels. In smaller coronary vessels, there is also evidence for smooth muscle cell proliferation (Sheppard 2011; Elleder 2003). Globotriaosylceramide metabolites, including de-acylated Gb3 and globotriaosylsphingosine, appear to stimulate vascular smooth muscle cells and inhibit α-galactosidase A activity (Barbey et al. 2006a; Aerts et al. 2008).

The carotid, brachial and aortic intima-media thickness is increased compared to normal controls in the absence of atherosclerotic plaques (Barbey et al. 2006b; Kalliokoski et al. 2006). Brachial artery flow-mediated dilatation is also impaired (Kalliokoski et al. 2006). Aortic dilatation at the sinus of Valsalva is common, affecting 32.7% of males and 5.6% of females and aortic aneurysms are described (Barbey et al. 2010). Aortic root dilatation may contribute to the development of aortic valvular regurgitation due to reduced aortic leaflet coaptation.

Chest pain is a common clinical symptom in patients even in the absence of significant LVH (Chimenti et al. 2008). The prevalence of angina in the Fabry outcome survey was 23% in females and 22% in males (Linhart et al. 2007). Whilst there have been case reports of patients with AFD developing premature coronary disease (Fig. 3F), there has been no definitive evidence of increased coronary atherosclerosis in this setting (Fisher et al. 1992; Chimenti et al. 2007). Most analyses are also limited by the absence of data on age and risk factor-matched control populations. In the Fabry outcome survey, there was a similar prevalence of myocardial infarct in women (1.6%) compared to men (0.8%) at baseline study entry in untreated (naïve to ERT) Fabry patients and overall there were relatively few events of coronary revascularization or myocardial infarction (Linhart et al. 2007). This supports data from another study demonstrating that during the natural history period of Fabry, only 2.7% of male patients and 1.5% of females experienced a myocardial infarct (Patel et al. 2011).

Intravascular ultrasound (IVUS) of the coronary arteries identifies evidence of diffuse coronary plaques which are hypo-echogenic and more likely to have lipid cores compared to control patients (Kovarnik et al. 2008). The latter finding may be accounted for by the higher lipid content in plaques due to glycosphingolipid accumulation in the endothelium of coronary arteries.

In a case report of a Fabry patient undergoing coronary artery bypass grafting (CABG) for multi-vessel coronary arterial disease, the internal mammary artery was found to be occluded at 1 year post-cardiothoracic surgery with recurrence of chest pain. This occlusion of a LIMA graft is unusual as they typically have a 98% patency rate at 1 year. Histological analysis of the LIMA artery showed diffuse glycosphingolipid storage in smooth muscle cells and the presence of fibrous tissue contributing to an abnormal arterial structure (Chimenti et al. 2007). This raised the hypothesis that arterial grafts in AFD patients may be more prone to occlusion through glycosphingolipid exposure, and venous conduits may be preferred. Other cases involving LIMA grafting in Fabry however have shown graft patency at 6 and 19 months post-operatively although histological analysis intra-operatively of the resected portion of the LIMA graft did demonstrate degeneration of the outer portion of the medial smooth muscle layer with intracellular vacuoles and collagen despite a normal endothelium and internal elastic lamina (Fisher et al. 1992; Osada et al. 2016).

Apart from macrovascular coronary arterial disease, AFD patients also have abnormal coronary microvascular function demonstrated by a lower hyperaemic myocardial blood flow on positron emission tomography (PET) imaging. ERT does not improve this parameter (Elliott et al. 2006). In another study of 15 patients, there was evidence of lower myocardial perfusion reserve (Kalliokoski et al. 2005). Microvascular ischaemia is believed to be a major mechanism of cardiac chest pain in the setting of AFD.

## Treatment of cardiac disease in AFD

### Enzyme replacement therapy

ERT aims to compensate for the reduced α-galactosidase levels and to reduce accumulation of glycosphingolipids in tissues. Two formulations of ERT are licenced, both administered as an intravenous infusion fortnightly, agalsidase-α (Replagal, Shire) and agalsidase-β (Fabrazyme, Sanofi-Genzyme). Agalsidase-α is derived from human fibroblasts whereas agalsidase-β is derived from Chinese hamster ovarian cells. Side effects of ERT include mild infusion reactions although serious side effects can include anaphylaxis. Patients may also develop IgG antibodies which might reduce treatment efficacy, and both formulations have similar antibody cross-reactivity (Lee et al. 2003).

ERT has been shown to reduce the severity of neuropathic pain, improve body weight and stabilise or improve renal creatinine clearance (Schiffmann et al. 2001; Baehner et al. 2003; Beck et al. 2004). From a cardiological perspective, ERT is associated with a small decrease in the QRS duration. Data on the impact of ERT on changes in left ventricular hypertrophy are conflicting with some studies showing a reduction in LV mass and improved myocardial function as assessed by systolic radial strain rate whereas others fail to show significant changes in ventricular wall thickness (Schiffmann et al. 2001; Baehner et al. 2003; Koskenvuo et al. 2008; Breunig et al. 2006). The benefits of ERT on cardiovascular symptoms and complications are less clear, in particular, in patients with established myocardial fibrosis or with extensive hypertrophy or significant valvular disease (Weidemann et al. 2009).

The Fabry outcome survey had reported that treatment with agalsidase-α resulted in stability in left ventricular mass index and mid-wall fractional shortening over a period of 5 years. This study also showed a sustained reduction in LV mass index and a significant increase in mid-wall fractional shortening after 3 years of ERT in those with LV hypertrophy at baseline evaluation (Mehta et al. 2009b). These changes were also associated with a reduction in T2 relaxation times on CMRI in all myocardial regions (Imbriaco et al. 2009).

Agalsidase-β treatment slows progression to a composite clinical outcome of cardiac, renal and cerebrovascular complications or mortality, although this was predominantly driven by reduction in renal complications including deteriorating renal function (Banikazemi et al. 2007). A recent pooled analysis of data in a systematic review suggested agalsidase-β was associated with a significantly reduced incidence of cardiovascular events when compared to treatment-naïve patients (El Dib et al. 2017).

In general, most data suggest that in advanced disease, ERT is unlikely to be transformative and in many cases, the cardiac structural phenotype progresses despite ERT (Mehta et al. 2009a). The presence of late gadolinium enhancement (LGE) or fibrosis on CMRI at baseline, before ERT institution, is associated with a lack of regression of LVH and worsening segmental myocardial function despite subsequent therapy (Weidemann et al. 2009).

### Chaperone therapy

More recently, oral chaperone therapy has become available for use in patients. Migalastat is a small molecule chaperone that reversibly binds to the active site of α-galactosidase and thereby stabilises mutant enzyme and promotes α-galactosidase-based catabolism of cellular products. In a study of 57 AFD patients randomised to ERT or migalastat, left ventricular mass index significantly decreased in patients on migalastat treatment (Hughes et al. 2016; Germain et al. 2016). Migalastat has been shown in case reports to reduce left ventricular hypertrophy and decrease LGE and associated cardiac serum biomarkers (TnT and NT-ProBNP) (Muntze et al. 2018). Further studies are required to assess the longer-term impact chaperone therapy has on the AFD cardiac phenotype.

### General management of cardiac disease

From a cardiac perspective, the majority of medications are for symptom relief and are not prognostic. Patients should have their cardiovascular risk profile managed with control and optimisation of blood pressure to minimise the risk of progressive left ventricular hypertrophy or nephropathy from suboptimally controlled hypertension.

In a cohort of patients on ERT, 30% of the study cohort was identified to be hypertensive at the time of study enrolment (Wu et al. 2010). Systolic blood pressure was also identified to be higher in AFD patients with LGE compared to those without, and the highest systolic blood pressures were identified to occur in a subgroup of patients with rapidly increasing LGE. This suggests that systolic BP can impact the rate of progression of Fabry’s cardiomyopathy (Kramer et al. 2015). In another analysis of 2869 Fabry registry patients naïve to ERT, multivariate analysis demonstrated both hypertension and LVH as risk factors that were strongly associated with the risk of a major adverse cardiovascular event (myocardial infarct, heart failure or cardiac-related mortality) in either gender with hypertension exhibiting an odds ratio of 4.5 in females (95% CI 1.6–12.3; *p* = 0.004) and 7.8 in males (95% CI 2.1–28.6; *p* = 0.002) on outcome (Patel et al. 2011).

Hypercholesterolaemia, another cardiovascular risk factor, is more prevalent in male AFD patients with cardiovascular events (42.9%) than in those without cardiovascular events (20.3%) (Patel et al. 2011). Statins are commonly prescribed in patients to help treat the associated hyperlipidaemia.

In the Fabry Registry, a prior history of smoking was common in both genders in those with cardiovascular events occurring before the commencement of ERT (44.7% of males and 38.6% of females with cardiovascular events) (Patel et al. 2011). Similar to the general population, the presence of cardiovascular risk factors including hypertension, renal dysfunction and smoking are associated with an increased incidence of cardiovascular events (Patel et al. 2011).

There are no large-scale randomised trial data of alternative medical agents in the setting of AFD. Management of LVOTO in the setting of Fabry is similar to that of patients with sarcomeric HCM and in accordance with ESC guidelines (Elliott et al -ESC Task Force 2014). Patients may be given β-blockers or non-dihydropyridine calcium antagonists (verapamil or diltiazem) for their negative inotropic and chronotropic action, and in the case of calcium antagonists, also for their negative lusitropic action. Care must be taken with β-blockers and non-dihydropyridine calcium antagonists which may potentiate bradycardia especially in the presence of conduction disease. Disopyramide may be considered as a second-line therapy in the management of LVOTO. In those with drug-refractory LVOTO, patients may be considered for more invasive therapy including right ventricular pacing with a short AV delay (DDDR pacing), alcohol septal ablation, septal myectomy or mitral valve intervention.

Diastolic dysfunction is treated with thiazide diuretics, mineralocorticoid antagonists (e.g. spironolactone) or loop diuretics (e.g. furosemide) to reduce LV filling pressures and pulmonary pressures. Higher doses of diuretics may also be needed in patients with fluid overload in the context of pulmonary or peripheral oedema especially in the setting of significant left ventricular systolic dysfunction. Coronary disease is treated identically to the general population.

In those with atrial fibrillation (AF), no established disease-specific guidelines are present, but in general, the threshold for anticoagulation is lower even with short paroxysms of AF given the increased risk of thromboembolism in the setting of structural or valvular heart disease (January et al. 2014; Yogasundaram et al. 2017). Antiarrhythmics such as sotalol or amiodarone may be used in Fabry patients although general principles of avoiding bradycardia and care with QTc prolongation are necessary. In patients with recurrent ventricular tachycardia or ICD ‘storms’, there are reported cases of successful combined endocardial and epicardial substrate VT ablation for treatment of ventricular arrhythmia (Higashi et al. 2011).

AFD patients may require permanent pacemaker implantation for anti-bradycardia indications in those with evidence of advanced conduction disease. A primary prevention implantable cardioverter defibrillator (ICD) may also be implanted as a prophylaxis to protect against the risk of sudden cardiac death in those thought to be at higher risk of such an event. Secondary prevention ICDs are implanted in those who have survived an aborted sudden cardiac death episode with successful resuscitation from ventricular dysrhythmia or in those with haemodynamically significant ventricular tachycardia.

In individuals that develop left ventricular systolic dysfunction, conventional heart failure medication and guidelines are followed with use of ACE inhibitors (or angiotensin receptor blockers), β-blockers and spironolactone (Ponikowski et al. 2016). There are only a few case reports on the role of cardiac resynchronization therapy (CRT) in patients with AFD and a ‘burnt out’ phenotype of severe LV systolic dysfunction. Concerns in the setting of AFD include that several studies have demonstrated poor response and outcomes from CRT in patients with greater scar burden and in particular those with extensive scar in the free wall of the LV (Leyva 2010). In those with symptoms of refractory heart failure, despite optimal medical therapy, advanced heart failure strategies such as orthotopic cardiac transplantation may be considered. Fabry’s development in the donor organ is unlikely to occur as the myocardial cells in the donor heart should possess normal levels of α-galactosidase A which should prevent Gb3 accumulation within the donor organ (Cantor et al. 1998).

### Potential future therapy

Gene therapy represents a potential curative treatment modality in AFD. As with various genetic disorders, research has been undertaken to assess the potential utilisation of gene therapy by recombinant adeno-associated virus-mediated gene transfer to correct the genetic defect in animal models of disease. Preliminary work has limited results but has suggested elevated α-galactosidase levels and reduced Gb3 deposition in various organs in transfected animals (Choi et al. 2010; Pacienza et al. 2012). The first in-human study of gene therapy via autologous stem cell transplantation using cells transduced via a lentivirus vector containing the ‘wild-type’ allele of *GLA* is currently underway (ClinicalTrials.gov 2018).

## Conclusions

Anderson-Fabry disease is a genetically transmitted condition that affects patients from a young age and substantially impairs their quality and quantity of life. Cardiovascular disease accounts for considerable morbidity in patients with AFD and in affected relatives, and a collaborative multi-disciplinary model of care for affected patients is essential to minimise morbidity and to optimise survival. Timely institution of ERT or chaperone therapy prior to the development of advanced cardiac disease or end-stage irreversible complications is essential to alter the natural course of disease. Conventional cardiovascular risk factor modification, including hypertension, is also important to minimise the risk of LVH progression and to avoid adverse cardiovascular events. In individuals with an established cardiac phenotype, management involves conventional medical therapy and regular monitoring enables pre-emptive intervention with pacemakers and primary prevention ICDs.
